# Release of free-volume bubbles by cooperative-rearrangement regions during the deposition growth of a colloidal glass

**DOI:** 10.1038/s41467-017-00428-4

**Published:** 2017-08-25

**Authors:** Xin Cao, Huijun Zhang, Yilong Han

**Affiliations:** Department of Physics, Hong Kong University of Science and Technology, Clear Water Bay, Hong Kong, 999077 SAR China

## Abstract

Vapor deposition can directly produce ultrastable glasses which are similar to conventional glasses aged over thousands of years. The highly mobile surface layer is believed to accelerate the ageing process of vapor-deposited glasses, but its microscopic kinetics have not been experimentally observed. Here we study the deposition growth kinetics of a two-dimensional colloidal glass at the single-particle level using video microscopy. We observe that newly deposited particles in the surface layer (depth, *d* < 14 particles) relax via out-of-cage diffusions of individual particles, while particles in the deeper middle layer (14 < *d* ≲ 100 particles) relax via activation of cooperative-rearrangement regions. These cooperative-rearrangement regions are much larger, more anisotropic and occur more frequently than cooperative-rearrangement regions in the bulk (*d* ≳ 100 particles) or after deposition. Cooperative-rearrangement regions move towards the surface and released free-volume bubbles at the surface, while the particles within cooperative-rearrangement regions move towards the bulk, resulting in a more compact bulk glass.

## Abstract (web summary)

Vapor deposition can produce ultrastable glasses similar to conventional glasses aged over thousands of years. Here authors study deposition growth kinetics of a two-dimensional colloidal glass and report relatively frequent occurrence of large and anisotropic regions of cooperative rearrangements at intermediate depths from the surface.

## Introduction

Conventional glasses formed from the quenching of liquids relax slowly to more stable structures via ageing. By contrast, vapor deposition can produce organic, polymeric and metallic glasses with extraordinary kinetic stability^1–5^. Such ultrastable glasses can have highly uniform amorphous structures^2^, unusually high^1, 2, 4^ densities, enhanced elastic moduli^2, 3, 6^ and highly anisotropic molecular orientations^5^. These properties are of significant interest in both practical material design and the theoretical understanding of the nature of glass transition. Experimental techniques such as differential scanning calorimetry^1^, neutron reflectivity^1^, dielectric measurements^7^, spectroscopic ellipsometry^5^, and wide-angle X-ray scattering^8^ have been applied to study vapor-deposited glasses. These studies emphasized the importance of surface mobility in the formation of ultrastable glasses. A highly mobile, liquid-like surface layer exists on the free surface of organic^9^ and polymeric^10–13^ thin-film glasses. This surface mobile layer is responsible for the reduced glass-transition temperature in polymer thin films^14^. In vapor-deposited glasses, similar surface mobile layer accelerates the rearrangement of newly deposited particles in a layer-by-layer fashion before they are buried into the bulk^1, 15–17^, but the microscopic relaxation kinetics remains to be confirmed experimentally.

Colloids are outstanding model systems for the study of glasses because the real-space trajectories of individual particles can be measured by video microscopy^18, 19^. Colloidal glass studies have mainly focused on the bulk properties or confinement effects^18, 20^, but have rarely explored free surfaces, i.e., vapor-glass interfaces. In this paper we study the relaxation near the free surface of a glass at the single-particle level for the first time by using colloids. In particular, we measured the kinetics during and after vapor deposition and found a mobile layer that was approximately 14 particles thick near the surface both during and after deposition. The newly deposited particles underwent frequent out-of-cage motion in the surface layer until they were buried into the bulk, which experimentally confirms the efficient relaxations via a surface mobile layer in the deposition growth of glasses^1, 15, 16^. Interestingly, we observed a middle layer (14 < *d* ≲ 100 layers of particles) which relaxes via the emergence of many large cooperative-rearrangement regions (CRRs). These CRRs propagated to the free surface, releasing free volumes to the vapor phase to give a more compact deposited glass.

## Results

### Experiment

**Fig. 1:**
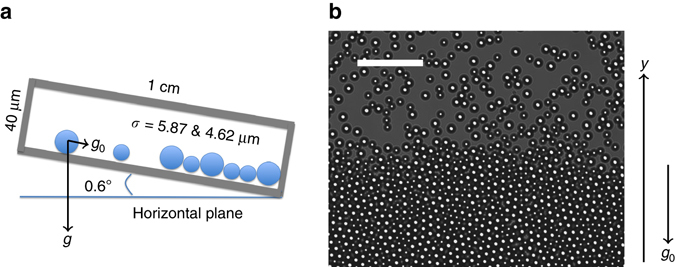
A binary colloidal glass monolayer formed by vapor deposition (Supplementary Movie 1). **a** Schematic of the sample cell. **b** The colloidal glass grew along the *y* direction by vapor deposition under gravity. *Scale bar*: 50 μm

We used a 45%:55% mixture of charge stabilized poly(methyl methacrylate) (PMMA) spheres with diameters *σ* = 4.62 and 5.87 μm in an aqueous suspension. The 2 cm × 1 cm × 40 μm sample cell was slightly tilted at an angle of approximately 0.6°, as shown in Fig. 1a. PMMA spheres with diameters *σ* = 4.62 and 5.87 μm and a mass density of 1.18 g cm^−3^ have gravitational heights *k*_B_ *T*/(mg) = 0.044 and 0.021 μm respectively, where mg is the buoyant weight, *k*_B_ is the Boltzmann constant and room temperature *T* = 295 K. Consequently, they settled on the substrate with negligible motions along the *z* direction and slowly drifted towards the lower end of the sample cell as shown in Fig. 1 and Supplementary Movie 1. The clear-cut solid-vapor interface propagated slowly at a speed of ${\nu _y} = 0.0112\,{\rm{\mu m}}\,{{\rm{s}}^{ - 1}} = 0.0386 \bar \sigma \cdot \tau _0^{ - 1}$ towards the vapor phase (Supplementary Fig. 1), where $\bar \sigma = 5.25\,{\rm{\mu m}}$ is the average diameter of spheres and *τ*_0_ = 18.1 s is the time needed for a particle in the vapor phase to diffuse a distance $\bar \sigma$.

### The surface is more mobile

**Fig. 2:**
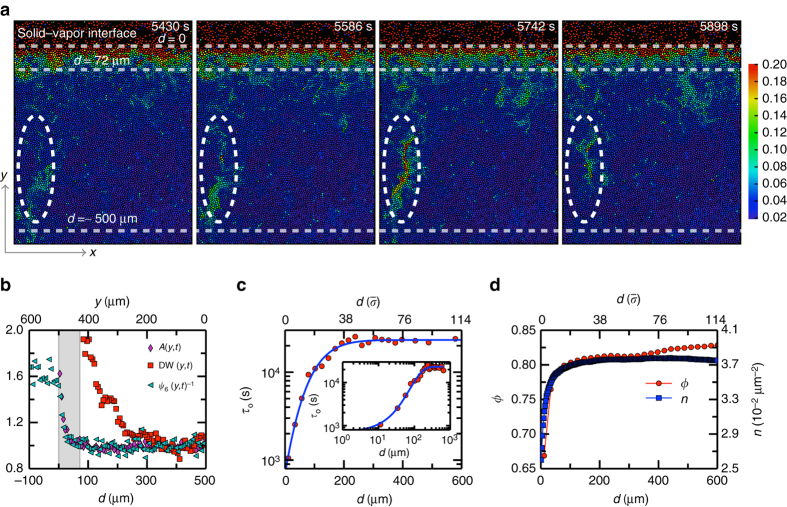
Surface profiles of structural and dynamic properties during vapor deposition. **a** Surface dynamics of the deposited monolayer glass from 5430 to 5898 s during growth. *Colors* represent the value of the DW factor of each particle. *d* = 0 marks the glass-vapor interface. $0 < d < 72\,\mu {\rm{m}} = 14\,\bar \sigma$ represents the highly mobile surface layer. In the middle layer of 72 μm < *d* ≲ 500 μm, many clusters of large-DW-factor particles, i.e., CRRs, emerged. The *dashed ellipses* show the evolution of a CRR. The bulk at *d* ≳ 500 μm has similar DW values as the middle layer, but much rarer and smaller CRRs. **b** Average area spanned by Voronoi polygons *A*(*y*, *t*), DW factor DW(*y*, *t*) and inverse of the crystalline order *ψ*_6_(*y*, *t*)^−1^ normalized by the bulk values as a function of depth *d*. The difference between their profiles defines a surface mobile layer at 0 < *d* < 72 μm marked by the *gray region*. At the boundaries of the *gray region*, DW and *A* are twice their corresponding bulk values. $\psi _6^{ - 1}$follows a similar profile as *A*. The curves are averaged over 100 frames with uniform time steps from 5470 s to 6250 s in Supplementary Movie 1. During such a time period, the surface propagated for about 1.7 particle diameters. **c** Profile of the mean out-of-cage time *τ*_o_(*d*) averaged over all frames. The fitting curve ${\tau _{\rm{o}}}\left( d \right){\rm{/}}{\tau _0} \sim {\left( {{\tau _{{\rm{bulk}}}}{\rm{/}}{\tau _0}} \right)^{f\left( {\left( {d - {d_0}} \right){\rm{/}}\xi } \right)}}$, where $f\left( {\sqrt 2 x} \right) = {\rm{erf}}\left( x \right) + 2x\,{\rm{exp}}\left( { - {x^2}} \right){\rm{/}}\sqrt \pi - 2{x^2}\left( {1 - {\rm{erf}}\left( x \right)} \right)$. erf(*x*) is the error function; *τ*_0_ = 18.1 s is the molecular time scale in vapor phase; *τ*_bulk_ = 2.3 × 10^4^ s is the mean *τ*_o_ in the bulk; *d*_0_ is the shift of the surface position and *ξ* is the length scale of CRRs^46^. *Inset*: The log-log plot. **d** Profiles of the area fraction *ϕ* and the particle number density *n* averaged over all frames. The averaged *ϕ* = 0.813 ± 0.007 in the middle layer and 0.827 ± 0.004 in the bulk

For each particle *i* at time *t*, we characterized its local density by its inverse Voronoi area 1/*A*_*i*_(*t*), the local sixfold crystalline order by *ψ*_6*i*_(*t*) and the strength of local dynamics by the Debye–Waller factor DW_*i*_(*t*) (see “Methods” section for details). The local dynamics is shown in Fig. 2a. Averaging these quantities over the *x* direction yields their profiles along the *y* direction as shown in Fig. 2b and Supplementary Movie 2. *A*(*y*, *t*) and DW(*y*, *t*) varied considerably at different depths, and the difference of these profiles reflects a surface mobile layer. We define the surface mobile layer whose *A*(*y*, *t*) is less than twice of the bulk value while DW(*y*, *t*) is greater than twice of the bulk value. The measured thickness of the surface mobile layer averaged over all frames is ${d_{{\rm{mob}}}} = 72 \pm 9\,{\rm{\mu m}} = 14 \pm 2 \,\bar \sigma$. This value is comparable to the atomic-layer thickness of the surface mobile layer measured in a thin-film polystyrene glass at the glass-transition temperature^14, 21^.

The relaxation time of a glass is usually characterized by the decay time of the intermediate scattering function *F*_s_(*q*, *t*). We measured *F*_s_(*q*, *t*) at different depths (Supplementary Fig. 2) and observed an obvious trend that relaxation was faster near the surface. Since the surface was shifting during the deposition and different depths are associated with different relaxation rates, the curves in Supplementary Fig. 2 may not reflect the real relaxation. Therefore, we characterized the structural relaxation near the surface by the mean out-of-cage time *τ*_o_, i.e., the time interval between two consecutive out-of-cage events for a particle (see “Methods”section for details). The measured *τ*_o_ is much shorter in the surface layer (Fig. 2c), reflecting more frequent out-of-cage events (Supplementary Movie 1). Each particle stayed in the surface mobile layer for approximately *d*_mob_/*ν*_*y*_ = 6.4 × 10^3^ s before it was buried into the middle layer, thus it experienced 5–10 out-of-cage events in the mobile layer since ${\tau _{\rm{o}}} \simeq {10^3}$ s. *τ*_o_ is ∼2.0 × 10^4^ s in the middle layer and the bulk (Fig. 2c), which agrees with the measured structural relaxation time *τ*_s_ = 1.4 × 10^4^ s in the bulk (Supplementary Fig. 2). When a particle is leaving the cage formed by its neighbors, the system is exploring the phase space from one local free-energy minimum to another. Therefore 1/*τ*_o_ is a measure of the rate of exploration in the phase space. During the deposition process, particles in the mobile layer quickly explore the phase space before they are buried into the bulk, forming an ultrastable glass layer-by-layer^1, 15, 16^. Figure 2c shows that the out-of-cage motion in the surface mobile layer is ~10 times faster than that in the middle layer, so the phase space exploration in the former is much faster. In addition, the fitting in Fig. 2c yields a length scale of CRR $\xi = 139\,{\rm{\mu m}} = 26\,\bar \sigma$, which agrees with the calculated maximum CRR length in *x* and in *y* directions (16 *σ* and 34 *σ* respectively). In Fig. 2d, the area fraction *ϕ* slightly increased from the middle layer to the bulk, while the number density *n* was almost constant. This is due to a slight change of the mixing ratio (4% more large spheres in the bulk). Vapor-deposited glasses are less stable when the temperature is too low or when the deposition rate is too high for the surface mobile layer to relax fully^15, 16^. Here we suggest that it is the thick slowly relaxing middle layer rather than the thin surface mobile layer, which determines the maximum deposition rate at which ultrastable glasses can form. If the middle layer is buried into the bulk before it is fully relaxed, the resulting glass will not be ultrastable.

### The middle layer relaxes via activations of CRRs

**Fig. 3:**
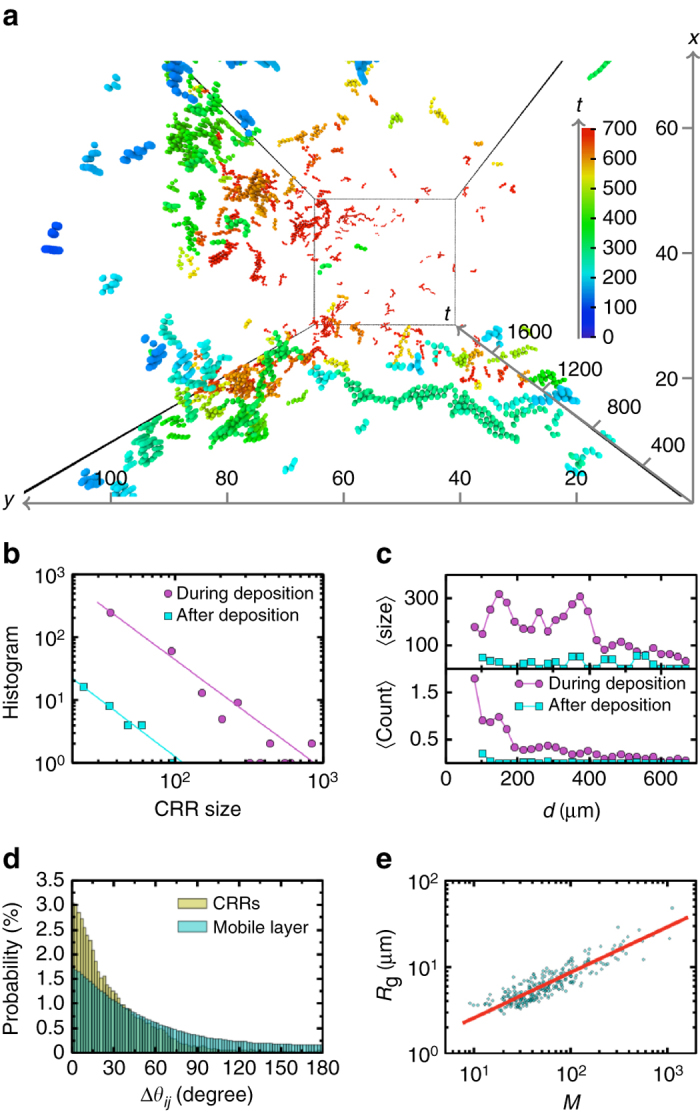
CRR morphologies. **a** CRRs in spacetime at *d* > 72 μm for a period of Δ*t* = 34,128 s during deposition. *Colors* indicate time. The unit for space is $\bar \sigma = 5.25\,{\rm{\mu m}}$ and the unit for time is *τ*_0_ = 18.1 s. **b** Histograms showing the size of CRRs, i.e., the particle number in spacetime, fitted by power laws with exponents *γ* = 1.74 ± 0.09 and *γ* = 1.79 ± 0.14 during and after deposition respectively. **c** The average CRR size and CRR count are much higher at 72 μm < *d* ≲ 500 μm than in the deeper bulk, indicating that a middle layer existed during deposition but vanished afterwards. **d** Histograms showing the angle Δ*θ*_*ij*_ between the displacements of particle *i* and its neighbor *j* during 156 s in all the CRRs during deposition and in the surface mobile layer. **e** Radius of gyration ${R_{\rm{g}}} = \mathop {\sum}\nolimits_i {{m_i}{{\left( {{{\overrightarrow {\bf{r}} }_i} - {{\overrightarrow {\bf{r}} }_c}} \right)}^2}{\rm{/}}M}$ of CRRs in spacetime during the deposition as a function of CRR mass $M = \mathop {\sum}\nolimits_i {{m_i}}$, where ${{\it{\overrightarrow {\bf{r}}}}_c} = {\Sigma _i}{m_i}{{\it{\overrightarrow {\bf{r}}}}_i}{\rm{/}}M$ is the position of the CRR’s center of mass in spacetime, *m*_*i*_ is the normalized area (i.e., *m*_*i*_ = 1 for small particles) of particle *i* and Σ_*i*_ is the sum over all particles in spacetime. The *red line* is the power-law fitting ${R_{\rm{g}}} \propto {M^{1/{d_{\rm{c}}}}}$ with the fractal dimension *d*_c_ = 1.91 ± 0.05. CRR mass is different from CRR size because a larger sphere has more mass

As a key concept in glass relaxation, CRRs have been intensively studied in the bulk^18, 22^, but rarely near surfaces. Bulk CRRs are like strings at high temperatures or in systems composed of repulsive particles^23^, while form compact domains at low temperatures or in systems composed of attractive particles^23, 24^. We measured the spacetime morphology (see details in “Methods” section) of CRRs as shown in Fig. 3a, which characterizes both the structure and dynamics of the local relaxations. DW > 0.12 particles are defined as CRR particles, while different threshold values yield similar results. Particles in the surface mobile layer moved rapidly and much less cooperatively, thus CRRs are not defined because otherwise the whole layer would be one huge CRR. The size of the CRRs in Fig. 3a follows a power-law distribution as shown in Fig. 3b. The power-law exponents in Fig. 3b are close to the power-law exponent 5/3 of the probability distribution of earthquake amplitudes, i.e., the Gutenberg–Richter law in seismology^25^. In fact, both CRRs and earthquakes are barrier-crossing processes involving the collective motions of densely packed materials.

We measured the density profile of the CRR count defined as the average number of CRR particles per frame at the instantaneous depth *d* (lower panel of Fig. 3c), i.e., the density profile of the colored particles along the *y* axis in Fig. 3a. Similarly, the CRR size profile defined as the average number of CRR particles at depth *d* weighted by their CRR size is shown in the upper panel of Fig. 3c. During deposition, many more CRRs were emerged in the middle layer than in the bulk (Fig. 3c), and they were also much larger in the middle layer, in agreement with the notion that the activation energy is lower near the surface^26^. After deposition, the packing fraction and CRRs of the middle layer became similar to those of the bulk(Fig. 3c and Supplementary Fig. 3), suggesting that the middle layer existed only during the deposition stage.

The collectiveness of particle motions in CRRs can be visualized in the wavy oscillation of DW factors in Supplementary Movie 2 and can be characterized by the angle Δ*θ*_*ij*_ between the displacements of neighboring particles *i* and *j*. The histograms of Δ*θ*_*ij*_ for CRR particles in the middle layer and in the surface mobile layer are shown in Fig. 3d. Δ*θ*_*ij*_ is distributed near 0° for CRR particles, indicating that neighboring CRR particles tended to move in a similar direction, i.e., string-like motion^27^ as shown in Fig. 3a. By contrast, Δ*θ*_*ij*_ for fast particles in the mobile layer in Fig. 2c show that their movements are much less cooperative. The CRRs in spacetime have fractal-like morphology with dimension *d*_c_ = 1.91 as shown in Fig. 3e. Fractal CRRs have been predicted in mean-field theory^23, 28^ and observed in colloid experiments^19, 24^ and computer simulations^29, 30^ in space, whereas we found that CRRs are still fractals in spacetime. In addition, string-like CRRs tended to be perpendicular to the surface when they were moving towards the surface, e.g., the long axes of the ellipses in Fig. 2a are perpendicular to the surface. This reflects the free-surface-induced symmetry breaking in both CRR structure and dynamics. The high-kinetic energy particles in vapor-deposited glasses can similarly form strings perpendicular to the surface, although these strings of hot particles are distinct from CRRs and only penetrate the surface layer of several particles^17^.

### A bubble-like picture of CRR

**Fig. 4:**
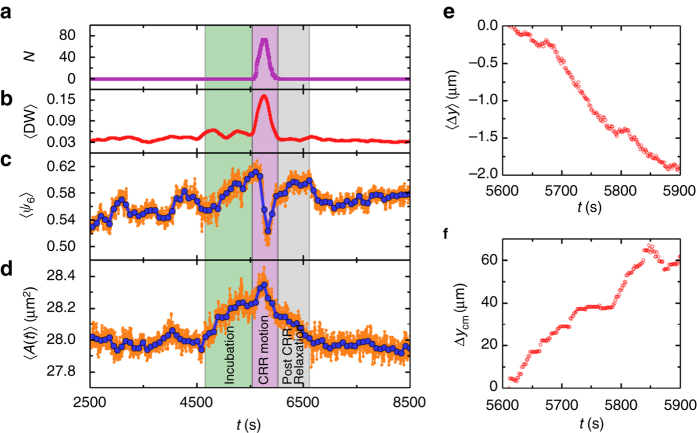
Evolution of the CRR in Fig. 2a. **a** Particle number *N*, **b** DW factor, **c** orientational order parameter 〈*ψ*_6_〉, and **d** Voronoi cell area 〈*A*〉. 〈〉 represents the average over all 80 particles that have ever participated in the CRR. The *blue curves* in **c**, **d** are smoothed over 50 bins (i.e., 78 s) of the raw data (*orange curve*). **e** Average displacement 〈Δ*y*〉 of the CRR particles as a function of *t*. Δ*y* is the displacement relative to the position at 5610 s. **f** Displacement of the center of mass Δ*y*_cm_ of the CRR relative to the position at *t* = 5610 s

The typical evolution of an individual CRR is shown in Fig. 4. Figure 4a–d reveal three stages of a CRR near the surface which have not been reported in previous CRR studies in bulk^23, 24^. In the initial incubation stage, 〈*A*〉 increased by 0.2 μm^2^ and 〈*ψ*_6_〉 increased slowly, while 〈DW〉 maintained a low value but fluctuated more strongly before an 80-particle CRR (Fig. 4a) emerged. This 80-particle CRR region absorbed Δ*A*_total_ = 0.2 μm^2^ × 80 = 16 μm^2^ of empty space from ambient regions. This amount of free volume is equivalent to 95% of the area of a small particle or 59% of the area of a large particle. This free volume triggered the collective motion, i.e., the CRR stage. The increased crystalline order (Fig. 4c) made more efficient use of the space, which also helped to make room for the rearrangement. In the CRR stage, 〈DW〉 developed a sharp peak (Fig. 4b) with a similar shape to *N*(*t*) (Fig. 4a). Meanwhile 〈*A*〉 rose to the maximum and 〈*ψ*_6_〉 developed a sharp trough, indicating that the CRR was accompanied by a minimum local density and maximum local disorder. In the third stage, the dynamic quantity 〈DW〉 relaxed immediately back to the equilibrium value, corresponding to the vanishing of the CRR. However, 〈*A*〉 equilibrated slowly in 500 s. The observed three stages for a CRR reflect the evolution of structure and dynamics during a barrier-crossing process from one inherent structure to another. The three stages with their similar features have been observed for most of the large CRRs in the middle layer, but are difficult to resolve in bulk CRRs because of their small size. Note that the density decrease in the incubation stage is not necessary for the CRR to emerge as long as the free volume is large enough (see Supplementary Fig. 4 for more demonstrations).

Interestingly, we found that particles in a CRR tended to drift towards the bulk, while the center of mass of the CRR propagated towards the surface. These behaviors are shown in Fig. 4e, f for an individual CRR and in Supplementary Fig. 5 for the ensemble average over all CRRs. A CRR can be viewed as a bubble of free volume since it is less dense than the ambient region (Fig. 4d). We observed that a free surface could attract bubbles within a depth *d* ≲ 100$\bar \sigma$ and caused these bubbles to float towards the surface. Eventually the bubbles were released into the vapor phase via CRRs, rendering a more compact bulk glass as observed in Fig. 2d. By contrast, bulk CRRs propagated randomly and could not change the mesoscopic density of the glass. This picture is similar to the free-volume diffusion model in describing the ageing of PMMA/silica nanocomposites^31^.

Small free volumes between particles in the middle layer can hardly migrate towards the free surface individually. However, when they nucleated and triggered with a CRR motion, their propagation towards the surface was dramatically enhanced. The observed free-volume nucleation agrees with the free-volume picture of viscous liquid, which described that molecular transport occurs via the movement of molecules into voids when the voids are larger than some critical size^32^.

## Discussion

The two relaxation mechanisms during deposition consist of the early-stage particle diffusion in the surface mobile layer and the later-stage release of free volumes towards the surface via CRR in the middle layer. Since a free surface should naturally induce these two effects within different depths, we expect that they occur commonly in other vapor-depositing glasses. Note that the middle layer existed only during deposition. After deposition, the middle-layer regime became indistinguishable from the bulk, which indicates a well-equilibrated glass after the bubble-releasing relaxation. These behaviors were confirmed in our simulation of bidispersed disks with Weeks–Chandler–Andersen potential: a CRR-rich middle layer 10 ≲ *d* ≲ 100$\bar \sigma$ formed below the *d* ≲ 10$\bar \sigma$ diffusive surface mobile layer during deposition but disappeared quickly afterwards (Supplementary Movie 3) (see “Methods” section for details of the simulation). In addition, the free-surface effect to CRR has rarely been experimentally explored before. Here we showed that a typical surface CRR exhibits three evolution stages with distinct morphology and dynamics to bulk CRRs. These results cast light on the formation of ultrastable glasses and the effect of surfaces on glass relaxation.

## Methods

### Experimental details

We mixed PMMA spheres with the diameters of 4.62 ± 0.11 μm (microParticles GmbH PMMA-R-B1170) and 5.87 ± 0.14 μm (microParticlesGmbH PMMA-R-B1298) at a ratio of 0.45:0.55. Two pieces of 40 μm-thick paraffin films were sandwiched between a glass slide and a glass coverslip as spacers, forming a 20 mm × 10 mm × 40 μm channel. A 10 μL drop of colloidal suspension was placed at one end of the channel and pulled into the channel by the capillary force. The whole sample was sealed with an epoxy adhesive. Colloidal solution in a properly sealed sample can be kept for a few months without drying. The whole microscope including the sample cell on the microscope stage was placed on a rotational rack and tilted at an angle of 0.6° with respect to the horizontal. At such a small tilt angle, the vapor phase of PMMA particles with a uniform area fraction of *ϕ* = 23.8% slowly settled towards the lower end of the sample cell (Supplementary Movie 1and Fig. 1b) and formed a monolayer glass with a clear-cut glass-vapor interface. The deposition process lasted for about one week before all particles in the vapor phase were deposited into the solid phase. One experimental video (i.e., Supplementary Movie 1) was recorded at the interface about 3 days after the deposition has started. Another experimental video (i.e., Supplementary Movie 4) was recorded at the interface about 1 day after the deposition has finished. Both videos lasted for about 10 h in real time. After deposition, the entire solid phase was about 3000 μm in depth.

### Radical tessellation, Voronoi area, and local crystalline order

The traditional Voronoi tessellation can characterize the local density of a monodisperse sphere system, but not the local density of a dense binary system because the bisecting plane between two neighboring spheres may cut through a large sphere and cannot reflect the real cage associated with each particle. Consequently we used the radical Voronoi tessellation in the Voro++ library^33^. In the radical Voronoi tessellation, the radical plane is composed of the points with the same tangent length for the two neighboring spheres, i.e., the tangential line from the point to each sphere has the same length. Radical Voronoi tessellation avoids the intersection with spheres and keeps the main topological features of the traditional Voronoi tessellation. It has been well adapted in grain hindrance and segregation problems^34^. An example of the radical Voronoi tessellation of our two-dimensional (2D) binary system is shown in Supplementary Fig. 6. The inverse area 1/*A*_*j*_ and the number of edges *N*_*nj*_ of the radical Voronoi polygon of particle *j* give the local density and the number of neighouring spheres. The profile of the mean Voronoi area along the *y* direction shown in Fig. 2b is calculated as $A(y,t) = {\int} {A(x,y,t){\rm{d}}x} {\rm{/}}{\int} {{\rm{d}}x}$. The local crystalline order ${\psi _{6j}} = \mathop {\sum}\nolimits_{k = 1}^{{N_{nj}}} {{{\rm{e}}^{ - i \cdot 6{\theta _{jk}}}}{\rm{/}}{N_{nj}}}$, where *N*_*nj*_ is the number of neighbors of particle *j*, *θ*_*jk*_ is the angle of the bond between particle *j* and its neighbor *k* and *i* ^2^ = −1. Similarly, the *ψ*_6_ profile along the *y* direction in Fig. 2b is calculated as ${\psi _6}(y,t) = {\int} {{\psi _6}(x,y,t){\rm{d}}x} {\rm{/}}{\int} {{\rm{d}}x}$.

### Debye–Waller (DW) factor

The dynamics of particle *j* can be characterized by the modified DW factor^35^defined as ${\rm{D}}{{\rm{W}}_j}(t) = 2\sqrt {{{\left\langle {{\rm{MSD}}} \right\rangle }_\tau }} {\rm{/}}\left( {{\sigma _1} + {\sigma _2}} \right)$ = $2\sqrt {{{\langle {{{( {{{\overrightarrow {\bf{r}} }_j} - {{\langle {{{\overrightarrow {\bf{r}} }_j}} \rangle }_\tau }} )}^2}} \rangle }_\tau }} {\rm{/}}\left( {{\sigma _1} + {\sigma _2}} \right)$, where ${\left\langle {} \right\rangle _\tau }$ denotes the average over a time period (*t* − *τ*, *t* + *τ*). *τ* = 156 s is the time needed for the mean-square displacement (MSD) of bulk particles to reach the middle of the plateau of the curve (Supplementary Fig. 7). The plateau of MSD arises from the cage of neighboring particles. Hence the square root of the plateau of MSD corresponds to the cage size. Note that the plateau of MSD or intermediate scattering function is much less pronounced in 2D than in 3D due to the Mermin–Wagner fluctuations in 2D, but glasses still exist in 2D^36–38^. This choice of *τ* can clearly distinguish uncaged particles with a DW_*j*_ ≥ 0.12. The profile of the mean DW factor along the *y* direction is shown in Fig. 2a. Supplementary Fig. 8 shows that the DW factor can reveal the CRRs and hence the middle layer, but the local density cannot.

### Out-of-cage time

The self-part of the intermediate scattering function ${F_{\rm{s}}}(q,t) = \langle {\mathop {\sum}\nolimits_{j = 1}^N {{{\rm{e}}^{i\overrightarrow {\bf{q}} \cdot [ {{{\overrightarrow {\bf{r}} }_j}(t) - {{\overrightarrow {\bf{r}} }_j}(0)} ]}}} } \rangle {\rm{/}}N$ characterizes the structural relaxation in glassy systems^39^. *F*_s_(*q*, *t*) averaged over time is inappropriate when the surface is moving because it would mix different relaxation times at different depths. Therefore *F*_s_(*q*, *t*) is well-defined only in the deep bulk or after deposition where the relaxation time stays constant. Alternatively we define an out-of-cage time *τ*_o_ to characterize the structural relaxation rate during deposition. *τ*_o_ is defined as the time interval between two out-of-cage events, i.e., three of the nearest neighboring particles will have been replaced since the last out-of-cage event. Here we set the threshold to three neighbors because a particle has six neighbors on average in 2D and an out-of-cage event will replace at least half of those neighbors (Supplementary Fig. 9a). The modification of two neighbors only may reflect a cage dilation instead of an out-of-cage event as shown in Supplementary Fig. 9b. In Supplementary Fig. 10, the mean-out-of-cage time is fitted by the mode-coupling theory^40^ *τ*_o_ ~ (*ϕ*_c_ − *ϕ*)^−*γ*^ where the fitted glass-transition point *ϕ*_c_ = 0.824 ± 0.006. It is lower than the measured *ϕ* = 0.827 ± 0.004 in 500 μm < *d* < 600 μm, indicating that the bulk was a glass.

### Characterization of the cooperative-rearrangement region

When a particle is undergoing cooperative-rearrangement, its DW factor will increase significantly (see the peak in Supplementary Fig. 11). In the middle layer and the bulk, 0.112% of particles have a DW > 0.12 and 0.282% of particles have a DW > 0.10. We define a mobile particle as one whose DW > 0.12. These mobile particles formed CRRs below the surface mobile layer as shown in Supplementary Fig. 12. The critical value of ~0.12 is in accordance with the Lindemann criterion that a crystal melts when the mean amplitude of particle vibrations exceeds ~10% of the lattice constant^41^. We define CRRs as clusters of mobile particles in spacetime which can better reflect their time evolution. By contrast, the conventional CRR defined as a cluster of mobile particles in space^19, 24^ is a cross-section of the CRR in spacetime. The time period for a DW factor exceeding 0.12 at the peak (e.g., Supplementary Fig. 11) is defined as the CRR time of a particle. Two particles are involved in the same CRR if they are neighbors in space and their CRR times overlap. We only consider CRRs involving more than one particle. The threshold of DW = 0.12 or 0.10 in the definition of mobile particles yields similar results about CRRs as shown in Supplementary Fig. 13.

### Computer simulation

We performed Brownian dynamics simulation of the deposition processes shown in Supplementary Movie 3. A CRR-rich middle layer approximately 100 atomic layers thick emerged during deposition but disappeared afterwards, which agrees with our experimental observations. The simulation system consisted of a 50%:50% binary mixture of particles interacting with Weeks–Chandler–Andersen (WCA) potential *U*(*r*) = 4*ε*[(*σ*/*r*)^12^ − (*σ*/*r*)^6^ + 1/4]^42^ with *m*_A_ = 1.0, *m*_B_ = 2.0, *σ*_AA_ = 1.0, *σ*_BB_ = 1.3, *σ*_AB_ = 1.15, and ${\epsilon _{{\rm{AA}}}} = {\epsilon _{{\rm{AB}}}} = {\epsilon _{{\rm{BB}}}} = \epsilon = 100$. The simulations were conducted in *N* *·* *AT* ensemble (constant number of particles *N*, area *A* = *l*_*x*_ × *l*_*y*_, and temperature *T*) with *N* = 15,000 particles in a box measuring *l*_*x*_ = 130 in width and *l*_*y*_ = 650 in length. The periodic boundary condition was applied in the *x* direction. Gravity was along the *y* direction. The ground at *y* = 0 was a WCA wall and the end of the vapor side at *y* = 650 was a reflection wall. A time step of d*t* = 0.0025 was used in all simulations. The temperature was kept constant using a Nose–Hoover thermostat. After the vapor was fully equilibrated at *k*_B_ *T* = 0.02*ε* in the absence of gravity, the temperature was decreased to *k*_B_ *T* = 0.005$\epsilon$, and the gravitational weights ${G_{\rm{A}}} = 0.0025\,\epsilon \cdot \sigma _{{\rm{AA}}}^{ - 1}$ and ${G_{\rm{B}}} = 0.005\,\epsilon \cdot \sigma _{{\rm{AA}}}^{ - 1}$ were turned on along the −*y* direction. The deposition process lasted for about 10^7^ steps until all vapor particles had been deposited on the glass.

### Influence of stuck particles

There were about 2–3% particles stuck on the substrate which can be identified from particles trajectories in the vapor phase in Supplementary Fig. 14 and the blue particles in the vapor phase in Fig. 2a. Stuck particles affected the trajectories of the nearby mobile particles in the vapor phase, but barely affected their neighbors in glass because there was no flow and most non-stuck particles were fully caged and similar to stuck particles (Supplementary Fig. 14). After stuck particles in the vapor phase were buried into the middle layer, we found that the positions of many CRRs were unrelated to that of stuck particles, indicating that CRR behaviors were not dominated by stuck particles. Hence stuck particles should not affect the conclusions of the present work although they can affect the glassy dynamics and the glass-transition point^43–45^. In fact, a free surface should naturally attract CRRs in a certain depth toward the surface and release the free volume. The middle layer featured by strong CRRs only existed during the deposition process and vanished after the deposition. This conclusion should also not be affected by stuck particles because stuck particles were the same before and after the deposition. These results are further confirmed in our simulation, which contains no stuck particles.

### Data availability

The data that support the findings of this study are available from the corresponding author upon request.

## Electronic supplementary material

Supplementary Information

Supplementary Movie 1

Supplementary Movie 2

Supplementary Movie 3

Supplementary Movie 4

## References

[1] Swallen SF (2007). Organic glasses with exceptional thermodynamic and kinetic stability. Science.

[2] Singh S, Ediger M, de Pablo JJ (2013). Ultrastable glasses from in silico vapour deposition. Nat. Mater..

[3] Yu H-B, Luo Y, Samwer K (2013). Ultrastable metallic glass. Adv. Mater..

[4] Lin P-H, Lyubimov I, Yu L, Ediger M, de Pablo JJ (2014). Molecular modeling of vapor-deposited polymer glasses. J. Chem. Phys..

[5] Dalal SS, Walters DM, Lyubimov I, de Pablo JJ, Ediger M (2015). Tunable molecular orientation and elevated thermal stability of vapor-deposited organic semiconductors. Proc. Natl Acad. Sci. USA.

[6] Kearns KL, Still T, Fytas G, Ediger M (2010). High-modulus organic glasses prepared by physical vapor deposition. Adv. Mater..

[7] Yu H, Tylinski M, Guiseppi-Elie A, Ediger M, Richert R (2015). Suppression of *β* relaxation in vapor-deposited ultrastable glasses. Phys. Rev. Lett..

[8] Gujral A, O’Hara KA, Toney MF, Chabinyc ML, Ediger M (2015). Structural characterization of vapor-deposited glasses of an organic hole transport material with X-ray scattering. Chem. Mater..

[9] Zhu L (2011). Surface self-diffusion of an organic glass. Phys. Rev. Lett..

[10] Forrest J, Dalnoki-Veress K, Stevens J, Dutcher J (1996). Effect of free surfaces on the glass transition temperature of thin polymer films. Phys. Rev. Lett..

[11] Ellison CJ, Torkelson JM (2003). The distribution of glass-transition temperatures in nanoscopically confined glass formers. Nat. Mater..

[12] Priestley RD, Ellison CJ, Broadbelt LJ, Torkelson JM (2005). Structural relaxation of polymer glasses at surfaces, interfaces, and in between. Science.

[13] Chai Y (2014). A direct quantitative measure of surface mobility in a glassy polymer. Science.

[14] Ediger M, Forrest J (2013). Dynamics near free surfaces and the glass transition in thin polymer films: a view to the future. Macromolecules.

[15] Kearns KL, Swallen SF, Ediger M, Wu T, Yu L (2007). Influence of substrate temperature on the stability of glasses prepared by vapor deposition. J. Chem. Phys..

[16] Kearns KL (2008). Hiking down the energy landscape: progress toward the Kauzmann temperature via vapor deposition. J. Phys. Chem. B.

[17] Reid DR, Lyubimov I, Ediger MD, de Pablo JJ (2016). Age and structure of a model vapor-deposited glass. Nat. Commun..

[18] Hunter GL, Weeks ER (2012). The physics of the colloidal glass transition. Rep. Prog. Phys..

[19] Weeks ER, Crocker JC, Levitt AC, Schofield A, Weitz DA (2000). Three-dimensional direct imaging of structural relaxation near the colloidal glass transition. Science.

[20] Hunter GL, Edmond KV, Weeks ER (2014). Boundary mobility controls glassiness in confined colloidal liquids. Phys. Rev. Lett..

[21] Paeng K, Swallen SF, Ediger M (2011). Direct measurement of molecular motion in freestanding polystyrene thin films. J. Am. Chem. Soc..

[22] Donati C (1998). Stringlike cooperative motion in a supercooled liquid. Phys. Rev. Lett..

[23] Stevenson JD, Schmalian J, Wolynes PG (2006). The shapes of cooperatively rearranging regions in glass-forming liquids. Nat. Phys..

[24] Zhang Z, Yunker PJ, Habdas P, Yodh A (2011). Cooperative rearrangement regions and dynamical heterogeneities in colloidal glasses with attractive versus repulsive interactions. Phys. Rev. Lett..

[25] Braun O, Peyrard M (2013). Role of aging in a minimal model of earthquakes. Phys. Rev. E.

[26] Stevenson JD, Wolynes PG (2008). On the surface of glasses. J. Chem. Phys..

[27] Weeks ER, Weitz D (2002). Properties of cage rearrangements observed near the colloidal glass transition. Phys. Rev. Lett..

[28] Klein W, Leyvraz F (1986). Crystalline nucleation in deeply quenched liquids. Phys. Rev. Lett..

[29] Donati C, Glotzer SC, Poole PH, Kob W, Plimpton SJ (1999). Spatial correlations of mobility and immobility in a glass-forming Lennard-Jones liquid. Phys. Rev. E.

[30] Johnson G, Mel’cuk AI, Gould H, Klein W, Mountain RD (1998). Molecular-dynamics study of long-lived structures in a fragile glass-forming liquid. Phys. Rev. E.

[31] Cangialosi D, Boucher VM, Alegria A, Colmenero J (2011). Free volume holes diffusion to describe physical aging in poly (mehtyl methacrylate)/silica nanocomposites. J. Chem. Phys..

[32] Cohen MH, Turnbull D (1959). Molecular transport in liquids and glasses. J. Chem. Phys..

[33] Rycroft, C. *Voro++: a three-dimensional voronoi cell library in C++*. Report No. LBNL-1432E (Lawrence Berkeley National Laboratory, 2009). 10.1063/1.3215722 20059195

[34] Gervois A, Oger L, Richard P, Troadec JP (2002). Voronoi and radical tessellations of packings of spheres. Computational Science-ICCS.

[35] Larini L, Ottochian A, Michele CD, Leporini D (2007). Universal scaling between structural relaxation and vibrational dynamics in glass-forming liquids and polymers. Nat. Phys.

[36] Shiba H, Yamada Y, Kawasaki T, Kim K (2016). Unveiling dimensionality dependence of glassy dynamics: 2d infinite fluctuation eclipses inherent structural relaxation. Phys. Rev. Lett..

[37] Vivek S, Kelleher CP, Chaikin PM, Weeks ER (2017). Long-wavelength fluctuations and the glass transition in two dimensions and three dimensions. Proc. Natl Acad. Sci. USA.

[38] Illing S (2017). Mermin-Wagner fluctuations in 2D amorphous solids. Proc. Natl Acad. Sci. USA.

[39] Kob W, Andersen HC (1995). Testing mode-coupling theory for a supercooled binary Lennard-Jones mixture. ii. intermediate scattering function and dynamic susceptibility. Phys. Rev. E.

[40] Gotze W, Sjogren L (1992). Relaxation processes in supercooled liquids. Rep. Prog. Phys..

[41] Zheng X, Earnshaw J (1998). On the Lindemann criterion in 2D. Europhys. Lett..

[42] Weeks JD, Chandler D, Andersen HC (1971). Role of repulsive forces in determining the equilibrium structure of simple liquids. J. Chem. Phys..

[43] Cammarota C, Biroli G (2012). Ideal glass transitions by random pinning. Proc. Natl Acad. Sci. USA.

[44] Karmakar S, Parisi G (2013). Random pinning glass model. Proc. Natl Acad. Sci. USA.

[45] Gokhale S, Nagamanasa KH, Ganapathy R, Sood AK (2014). Growing dynamical facilitation on approaching the random pinning colloidal glass transition. Nat. Commun..

[46] Salez T, Salez J, Dalnoki-Veress K, Raphaël E, Forrest J (2015). A Cooperative strings and glassy interfaces. Proc. Natl Acad. Sci. USA.

