# Supplementary material for: Release of free-volume bubbles by cooperative-rearrangement regions during the deposition growth of a colloidal glass
Source: Nat Commun. 2017 Aug 25;8:362. doi: 10.1038/s41467-017-00428-4 (PMC5572473; doi:10.1038/s41467-017-00428-4)
Supplement: Supplementary file 1 — Supplementary Information [file 41467_2017_428_MOESM1_ESM.pdf]

Description of Supplementary Files

File Name: Supplementary Information
Description: Supplementary Figures

File Name: Supplementary Movie 1
Description: The deposition process corresponding to Fig.~2a. In the surface mobile layer, liquid-like particles frequently moved out of their cages. Under the mobile layer was a middle activation layer which contained large string-like CRRs. These CRRs propagated towards the free surface and released free volumes toward the surface.

File Name: Supplementary Movie 2
Description: Evolution of the profiles of the Voronoi area $A(d,t)$ and the DW factor $\textrm{DW}(d,t)$.The field of view was fixed during deposition, thus the depth d changed as the free surfacepropagated. At a small d, the mobile layer has a similar density to the bulk but its DW factors are much higher. The DW factor in the middle activation layer shows strong and correlated fluctuations, representing the collective motions of CRRs. The surface propagated outside the field of view at the end.

File Name: Supplementary Movie 3
Description: The deposition process in the simulation. A surface mobile layer comprising approximately 10-20 particle layers was present during and after deposition. A CRR-rich middle layer comprising approximately 100 particle layers was present during deposition but quickly disappeared afterwards.

File Name: Supplementary Movie 4
Description: After deposition, the CRRs appeared much less frequently with much smaller sizes. The movie was recorded one day after the one-week deposition process.

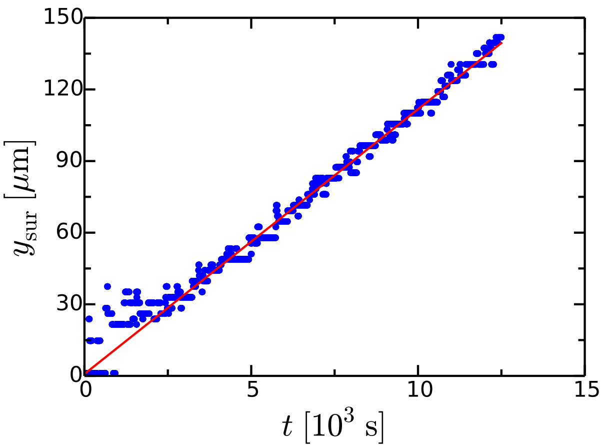

Supplementary Fig. 1. Position of the surface $y_{\rm sur}$ as a function of time t during vapour deposition. The slope of the linear fit (red line) yields the propagation speed $\nu = 0.0112$ μm $\cdot$ s^{-1}.

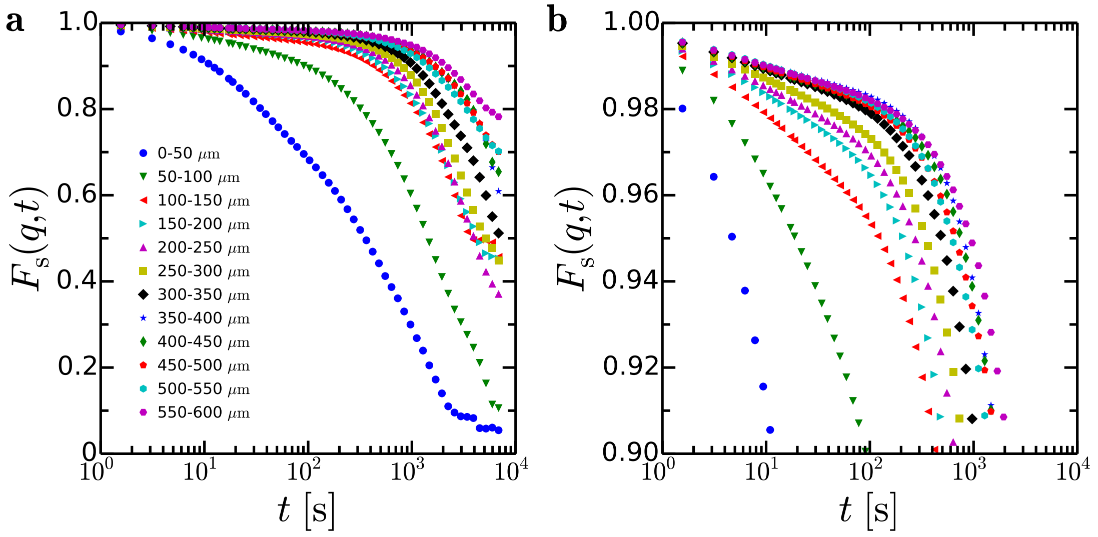

Supplementary Fig. 2. (**a**) The self-part of the intermediate scattering function $F_{\rm s}(q,t)$ at different depths. The particle trajectories used here are the same as those used in Supplementary Fig. 7. $q = 4\pi/(\sigma_1 + \sigma_2)$. The relaxation time is on the order of 10^4 s in the bulk. (**b**) The blow-up of (a).

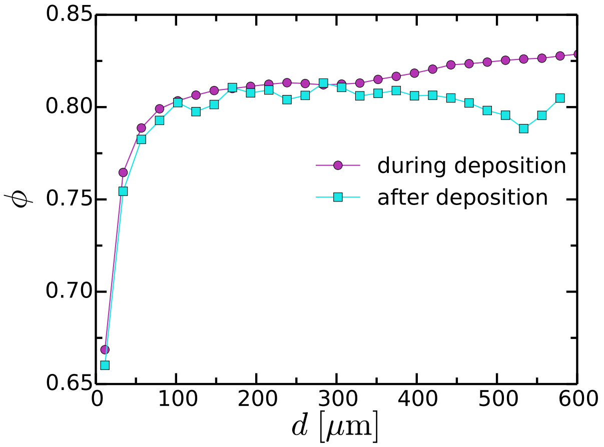

Supplementary Fig. 3. The area fraction profiles during the deposition and after the deposition. ϕ was nearly a constant at 100 μm $< d <$ 600 μm after the deposition.

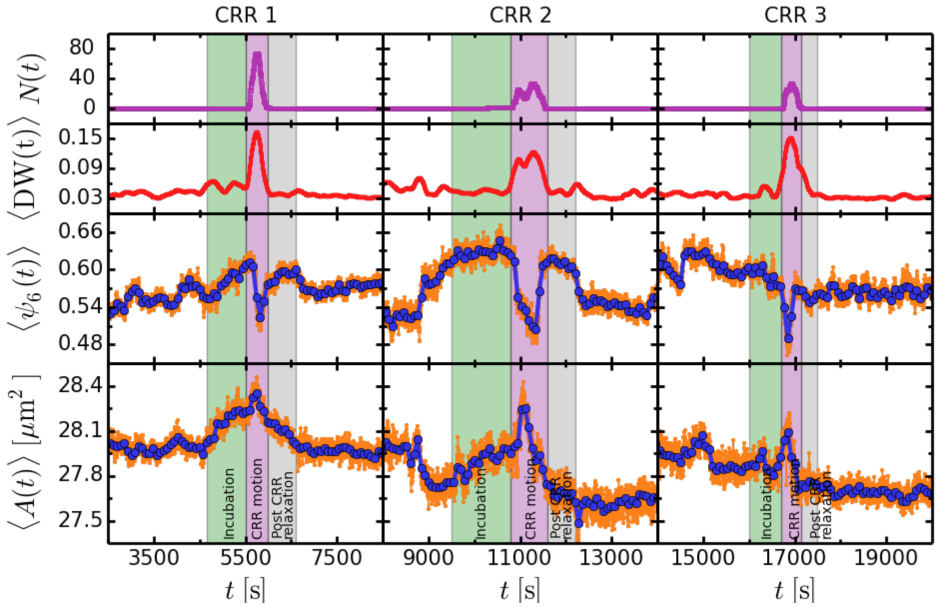

Supplementary Fig. 4. Time evolution of the number of CRR particles N, the DW factor, the crystalline order $\langle \psi_6 \rangle$ and the average Voronoi area $\langle A \rangle$ as a function of time for the three largest CRRs. The blue curves are smoothed over 50 bins (i.e. 78 s) of the raw data (the orange curves). $\langle \quad \rangle$ denotes the average over all particles in the CRR. The results suggest that CRRs are free-space bubbles (lower A than the surroundings) with a more disordered structure (lower ψ_6). During a CRR event, a free-space bubble is released toward the surface, effectively reducing the local density.

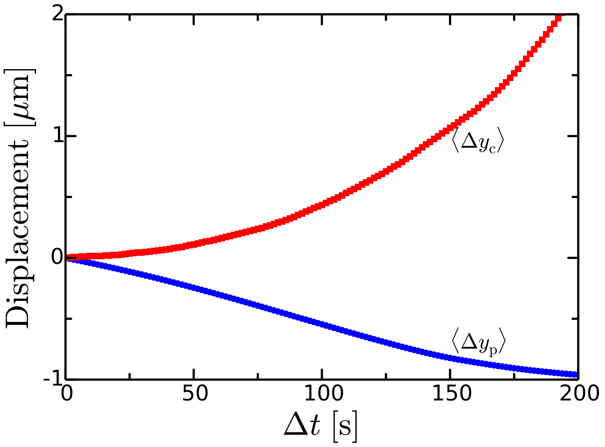

Supplementary Fig. 5. Displacements of CRR centers of mass $\langle \Delta y_c \rangle$ and CRR particles $\langle \Delta y_p \rangle$ averaged over all CRRs in spacetime. CRR centers of mass propagated toward the surface ($\langle \Delta y_c \rangle > 0$), while CRR particles moved into the bulk ($\langle \Delta y_p \rangle < 0$).

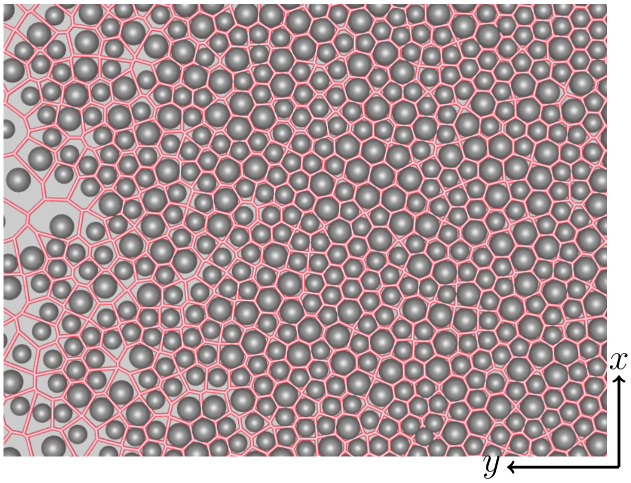

Supplementary Fig. 6. Radical Voronoi tessellation for the bidispersed monolayer near the glass-vapour interface.

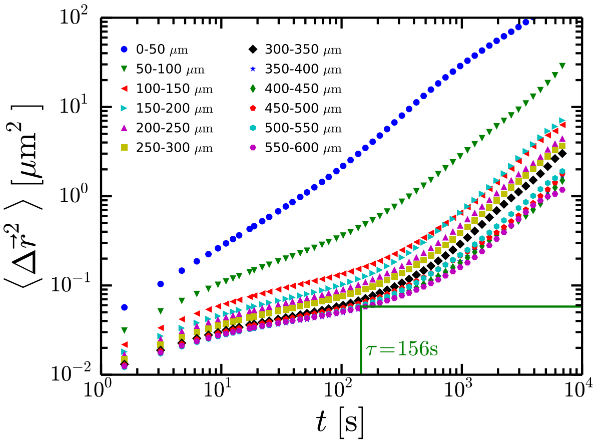

Supplementary Fig. 7. Mean-square displacements at different depths from 8410 s to 16220 s in Supplementary Movie 1. The position of the surface propagated 87 μm during this time period. Therefore the depth of each curve has a drift. Nevertheless the overall trend clearly shows that the diffusion is much faster near the surface. $\tau = 156$ s at the plateau of the bulk is used to calculate the DW factor.

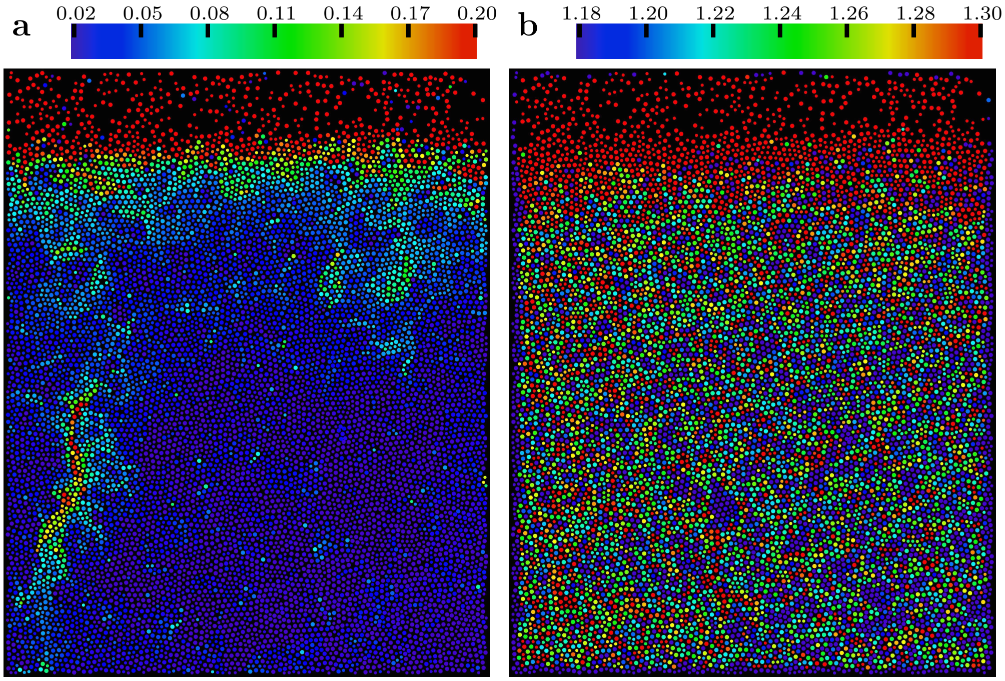

Supplementary Fig. 8. The same sample image coloured according to the DW factor (**a**) and the Voronoi area (**b**). The Voronoi areas of large and small particles are normalized by their corresponding particle areas. The surface mobile layer can be seen in both versions where the dynamics is fast in (a) and the density is low in (b). However the middle layer featuring large anisotropic CRRs can only be seen in (a) because CRRs have much stronger dynamics but only slightly lower densities than the ambient regions. Images in both (a) and (b) are 500×620 μm^2 in dimension.

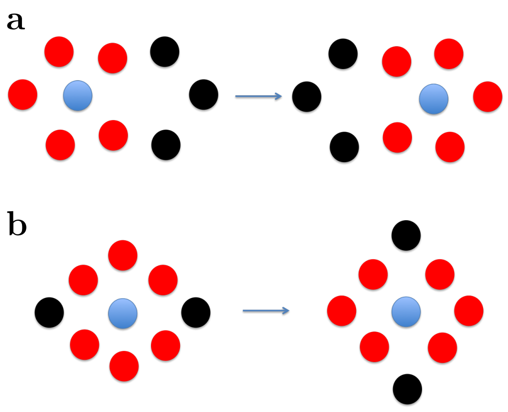

Supplementary Fig. 9. (**a**) When a particle leaves its original cage, three of its neighbours are replaced. Red particles represent the nearest neighbours. (**b**) A cage dilation replaces two neighbours of a particle, but the particle remains in the original cage.

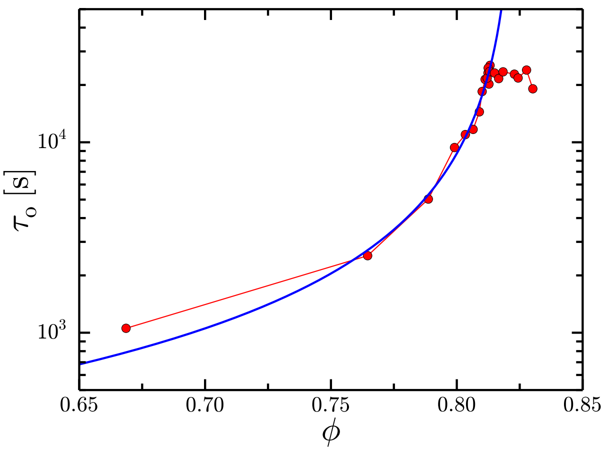

Supplementary Fig. 10. The mean out-of-cage time fitted by $\tau_o \sim (\phi_c - \phi)^{-\gamma}$ at $\phi < 0.814$. The fitted $\phi_c = 0.824 \pm 0.006$.

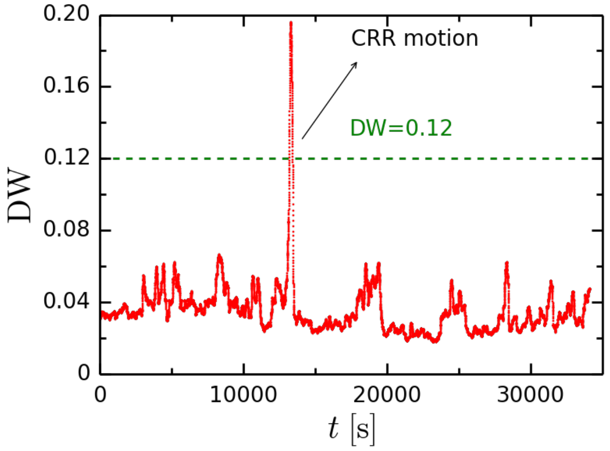

Supplementary Fig. 11. Typical evolution of the DW factor of a particle. The spike indicates that the particle was undergoing CRR.

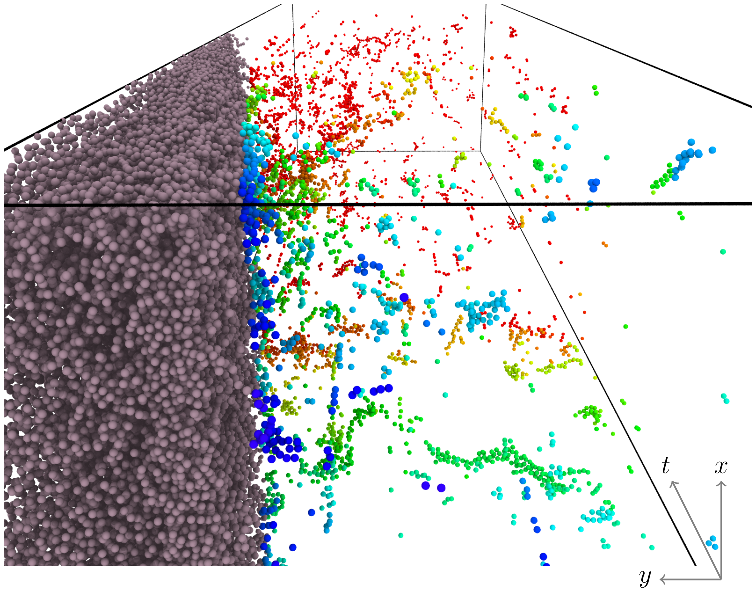

Supplementary Fig. 12. Particles with DW ≥ 0.12 are shown in spacetime. The colours indicate time order. These particles tended to form clusters, i.e. CRRs, suggesting that cooperative rearrangement is the dominant way of relaxation under the surface mobile layer. Gray particles are in the surface mobile layer or in the vapour phase, where CRRs are not well defined. Fig. 3a in the main text only shows the middle layer and the bulk region without the gray particles. Image dimension in (x, y, t) spacetime is $500 \times 620 \times 34000$ $\mu\text{m}^2 \cdot s$ in dimension.

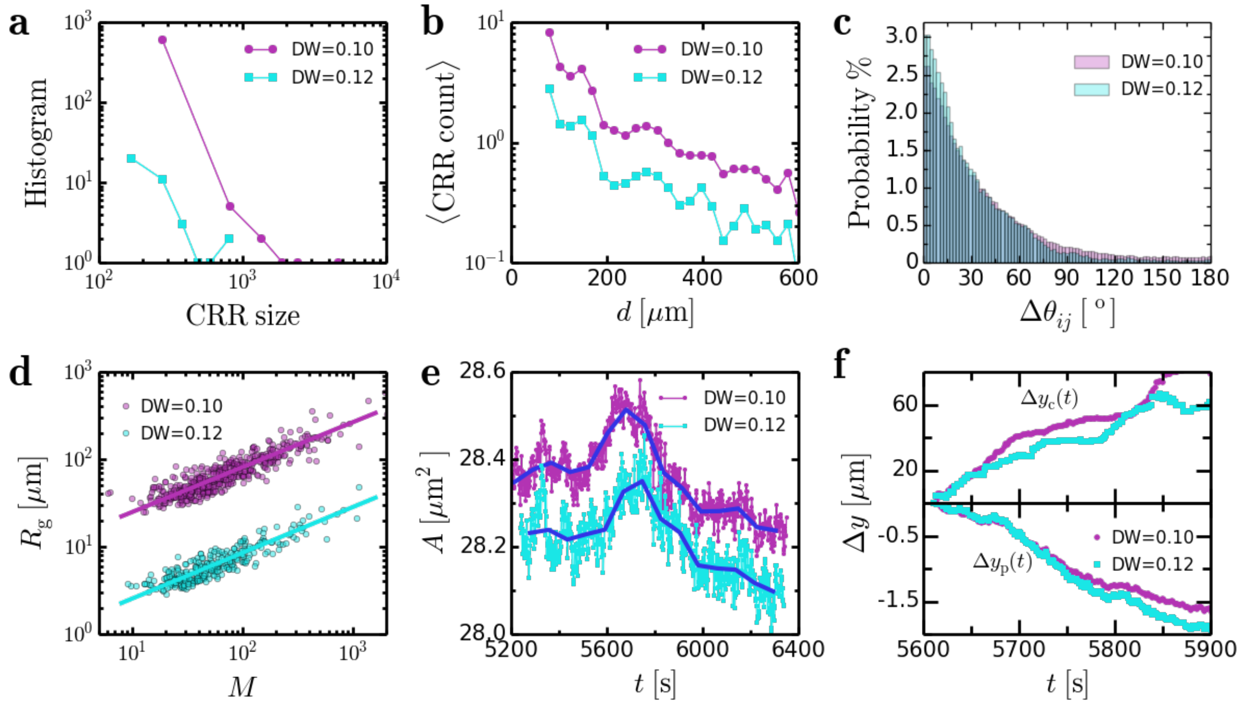

Supplementary Fig. 13. CRR particles identified from two different thresholds of the DW value, 0.10 and 0.12, exhibit similar CRR properties. (**a**) Histograms of the CRR size. (**b**) Average number of CRRs as a function of depth d. (**c**) Probability distribution of the angle difference between a particle's displacement and its neighbour's displacement during a period of 156 s. (**d**) Radius of gyration R_{g} as a function of CRR mass. The power-law fittings (red lines) give the fractal dimensions of 1.95 and 1.91 for DW values of 0.10 and 0.12 respectively. R_{g} for a DW value of 0.10 is multiplied by 10 to avoid overlap. (**e**) Time evolution of the average Voronoi area A for the particles in the CRR shown in Fig. 2a in main text. The blue curves are smoothed over 50 bins (i.e. 78 s) of the raw data (the purple curve and cyan curve). (**f**) The center of mass of the CRR in Fig. 2a in main text propagated toward the surface, i.e. the displacement $\Delta y_{\mathrm{c}} > 0$ (upper panel), while the CRR particles moved into the bulk, i.e. $\Delta y_{\mathrm{p}} < 0$ (lower panel).

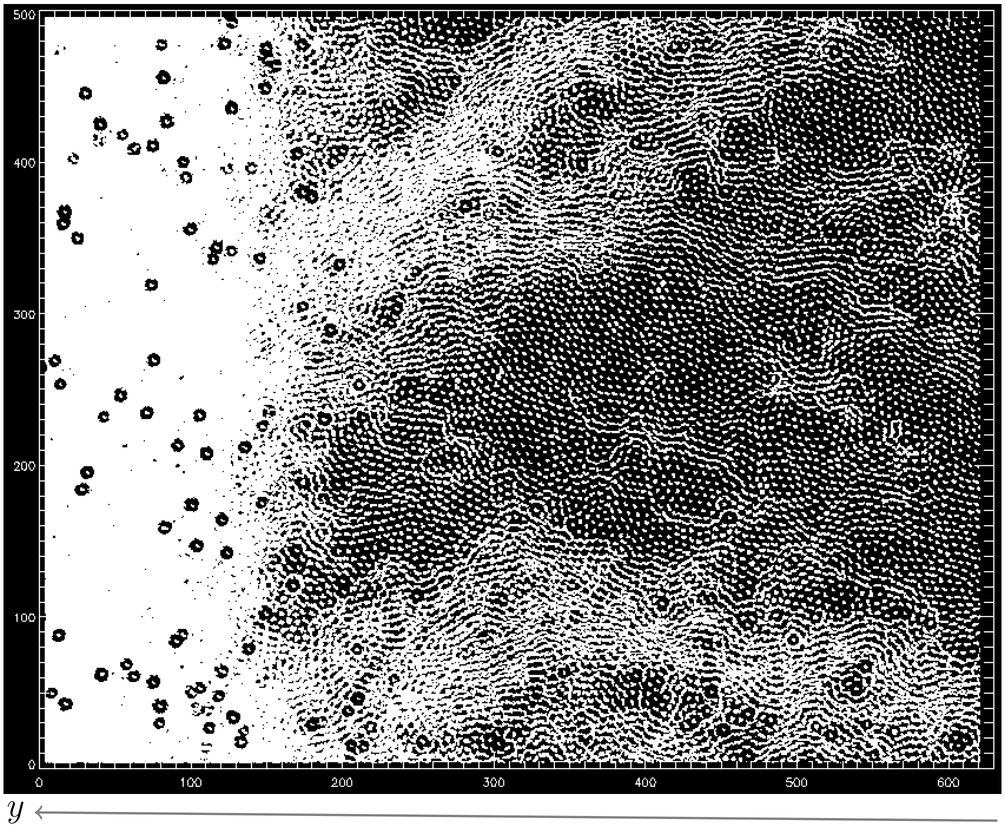

Supplementary Fig. 14. The superposition of particles' positions (white points) in the 500 × 600 μm^2 area in Supplementary Movie 1 (about 20000 frames or 10-hour video) recorded on the third day during a one-week deposition process. The y axis represents the growth direction. The vapor phase is white because the drift and Brownian motions made particles' trajectories explored all the vapor area. In the glass, most particles were fully caged without participating any CRR over the entire period of 10 hours, hence they formed disconnected white spots. Each white spot consists of about 20000 tiny white dots of the particle positions. White stripes show the regions that had ever involved in a CRR. A dark ring in the vapor region corresponds to a particle stuck on the substrate. Dark rings cover about 2-3% vapor area, hence about 2-3% particles were stuck.
